# G9a inhibits MEF2C activity to control sarcomere assembly

**DOI:** 10.1038/srep34163

**Published:** 2016-09-26

**Authors:** Jin Rong Ow, Monica Palanichamy Kala, Vinay Kumar Rao, Min Hee Choi, Narendra Bharathy, Reshma Taneja

**Affiliations:** 1 Department of Physiology, Yong Loo Lin School of Medicine, National University of Singapore, 117597, Singapore; 2 NUS Graduate School for Integrative Sciences and Engineering, National University of Singapore, 117456, Singapore

## Abstract

In this study, we demonstrate that the lysine methyltransferase G9a inhibits sarcomere organization through regulation of the MEF2C-HDAC5 regulatory axis. Sarcomeres are essential for muscle contractile function. Presently, skeletal muscle disease and dysfunction at the sarcomere level has been associated with mutations of sarcomere proteins. This study provides evidence that G9a represses expression of several sarcomere genes and its over-expression disrupts sarcomere integrity of skeletal muscle cells. G9a inhibits MEF2C transcriptional activity that is essential for expression of sarcomere genes. Through protein interaction assays, we demonstrate that G9a interacts with MEF2C and its co-repressor HDAC5. In the presence of G9a, calcium signaling-dependent phosphorylation and export of HDAC5 to the cytoplasm is blocked which likely results in enhanced MEF2C-HDAC5 association. Activation of calcium signaling or expression of constitutively active CaMK rescues G9a-mediated repression of HDAC5 shuttling as well as sarcomere gene expression. Our results demonstrate a novel epigenetic control of sarcomere assembly and identifies new therapeutic avenues to treat skeletal and cardiac myopathies arising from compromised muscle function.

The myocyte enhancer factor 2 (MEF2) family of transcription factors regulates several aspects of skeletal muscle development and function^1^,^2^,^3^. In vertebrates, the MEF2 family consists of four members (MEF2A, MEF2B, MEF2C and MEF2D), each containing a highly conserved N-terminal minimal DNA-binding MADS box, the MEF2 domain that is responsible for dimerization and DNA-binding^4^, and the less conserved C-terminal transactivation domain^5^. MEF2 activity is regulated by several mechanisms. Class II histone deacetylases (HDACs) [HDAC4, −5, −7 and −9] are among the best characterized repressors of MEF2 function^6^. While lacking intrinsic HDAC activity, class II HDACs repress MEF2 activity via recruitment of co-repressors including class I HDACs [HDAC3/NCoR/SMRT]^7^ and histone methyltransferases [SUV39H1/HP1]^8^. Class II HDACs are known to shuttle in and out of the nucleus in response to various stimuli. In response to calcium signaling, class II HDACs are phosphorylated by calcium/calmodulin-dependent protein kinase (CaMK), resulting in their dissociation from MEF2 9 and subsequent 14-3-3 dependent export from the nucleus^10^. Protein kinase D (PKD) and the serine/threonine protein kinases MARK1 and MARK2 also phosphorylate class II HDACs at distinct sites to regulate nuclear/cytoplasmic shuttling^11^,^12^. In addition to activation by CaMK, the transcriptional activity of MEF2 is enhanced by additional mechanisms that include phosphorylation of the transactivation domain by the mitogen activated protein kinases (MAPK) p38^13^ and ERK5^14^ as well as association with the co-activator p300^15^. Since both co-repressors and co-activators associate with the MEF2 domain, class II HDACs block MEF2 activity by competing with p300 for binding to MEF2^16^. In addition, interaction with class II HDACs prevents MEF2 from being activated by the p38 MAPK pathway^9^ and this repression is lifted in the presence of calcium signaling.

Disruption of the single MEF2 gene in *Drosophila* impedes muscle development and results in reduced expression of structural genes^17^,^18^. In vertebrates, MEF2C is the first member to be expressed in the myotome, followed by MEF2A and MEF2D^19^. Interestingly, in mice, skeletal muscle-specific loss of only MEF2C impacts muscle development. Myofibers from MEF2C mutant mice develop normally during embryogenesis and do not show any defects in differentiation. However, the myofibers deteriorate soon after birth due to loss of sarcomere integrity^20^. MEF2C regulates expression of several structural and sarcomeric genes including myomesins, myozenins, myotilins, myosin light and heavy chain, sarcoglycans, and components of the troponin complex^3^,^20^. Similarly, studies in zebrafish have shown that MEF2C and MEF2D are required for sarcomere assembly and control expression of genes required for thick filament formation^21^.

Consistent with their central role in regulating MEF2 activity, class II HDACs regulate fiber type specific programs that are MEF2-dependent^22^,^23^. In addition, they mediate the repressive effects on MEF2-dependent structural and contractile genes *in vivo*^24^. As such, increased expression of class II HDACs in skeletal muscle are associated with congenital myopathies^25^ that display disruption of sarcomere assembly and reduced expression of MEF2 target genes such as Lmod3^26^. Another co-repressor that has been implicated in the regulation of muscle structural and sarcomere genes is G9a. G9a, also known as Euchromatic Histone-lysine N-methyltransferase 2 (EHMT2) or KMT1C, contains a C-terminal enzymatic Su(var)3–9, Enhancer of zeste and Trithorax (SET) domain which mediates repressive histone H3 lysine 9 di-methylation marks at euchromatic regions of the genome^27^,^28^,^29^. In embryonic stem cells, G9a exists in a hetero-dimer with its paralog, G9a-Like Protein (GLP), and notably, loss of G9a in mice results in embryonic lethality^30^. Interestingly, however, conditional knockout of G9a in postnatal neurons causes de-repression of muscle sarcomere genes, demonstrating its critical role as a repressor of the contractile machinery *in vivo*^31^. Although implicated in myogenesis^32^,^33^, whether G9a regulates sarcomere genes in skeletal muscle cells, and the mechanisms by which it does so have not been investigated.

In this study, we demonstrate that G9a represses sarcomeric genes and inhibits sarcomere assembly by impacting the HDAC5-MEF2 circuitry. Down-regulation of G9a expression or pharmacological inhibition of its methyltransferase activity in skeletal myoblasts results in the up-regulation of several MEF2-dependent structural and sarcomeric genes, including myomesins and myozenin. We demonstrate that G9a associates with MEF2C and blocks nuclear export of HDAC5, resulting in repression of MEF2C transcriptional activity. G9a occupancy and histone H3 lysine 9 di-methylation (H3K9me2) marks are apparent at MEF2 sites on sarcomeric gene promoters, and its over-expression results in sarcomere disorganization. Activation of calcium signaling in G9a over-expressing cells restores HDAC5 shuttling and MEF2C-dependent sarcomere gene expression. Together, these studies provide evidence that G9a forms a repressive complex with MEF2C and HDAC5 to epigenetically control sarcomere organization.

## Results

### Sarcomeric genes are regulated by G9a

**Figure 1:**
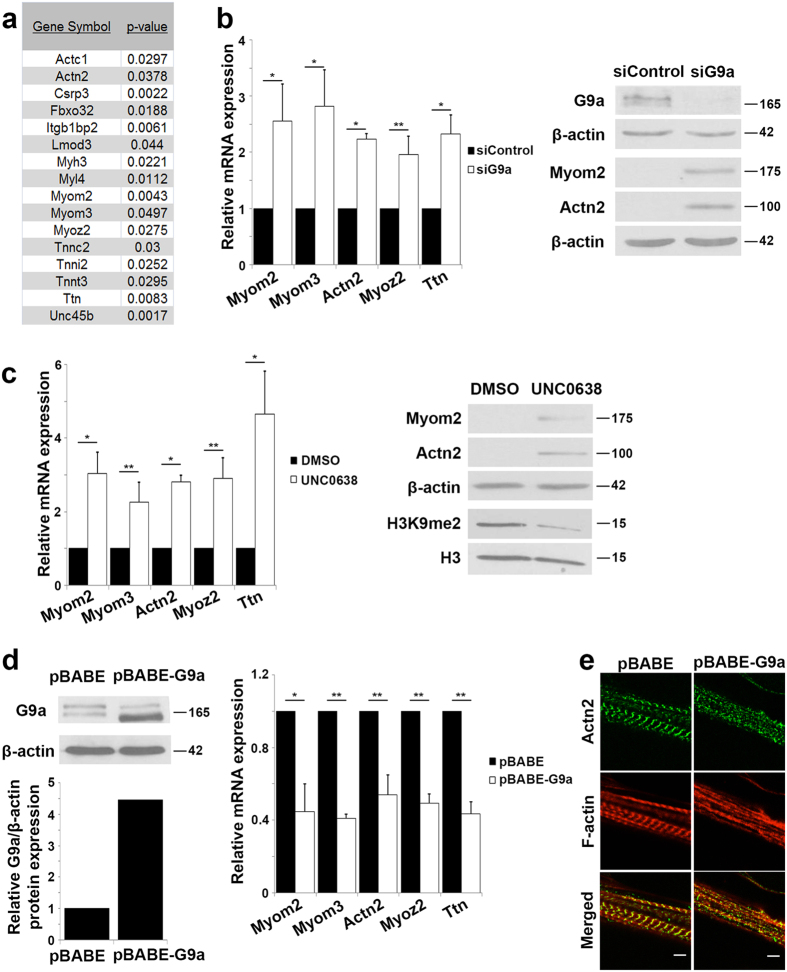
G9a represses sarcomere genes and assembly. (**a**) Partial list of sarcomere genes that are up-regulated in proliferating siG9a C2C12 myoblasts compared to siControl cells with corresponding *p*-values. The list is from data set GSE70039^34^. (**b**) C2C12 myoblasts transfected with scrambled siRNA (siControl) or G9a-specific siRNA (siG9a). The mRNA expression of Myom2, Myom3, Actn2, Myoz2 and Ttn was analyzed by qPCR. The expression in control cells was given an arbitrary value of 1. The fold change relative to control cells is shown. Lysates were analyzed for G9a, Myom2 and Actn2 expression by western blot. β-actin was analyzed as a loading control. (**c**) Undifferentiated C2C12 cells were treated with DMSO or UNC0638 for 48 hr. mRNA expression of sarcomeric genes in the presence or absence of UNC0638 treatment was examined by qPCR. Myom2 and Actn2 protein levels as well as global H3K9me2 levels were analyzed by western blot. β-actin and Histone H3 were used as loading controls. (**d**) C2C12 cells were transduced with empty retroviral vector (pBABE) or with pBABE-G9a. G9a expression was analyzed by western blot (upper panel). Densitometric analysis of the western blot showed ~4.5 fold higher expression of G9a in pBABE-G9a cells (lower panel). Sarcomeric gene expression in undifferentiated pBABE and pBABE-G9a cells was probed by qPCR. (**e**) pBABE and pBABE-G9a cells were differentiated for six days. Actn2 (green), a marker of the Z-disc, and F-actin (red) within the thin filament that form part of the sarcomere were analyzed by immunofluorescence. Confocal images were taken at 100X magnification with 3X focus. Scale bar: 5μm. Error bars indicate mean ± standard error of n = 3. Numbers indicate molecular weight of proteins.

Previous studies have shown that G9a regulates MEF2 transcriptional activity^33^ which plays an essential role in sarcomere assembly^20^,^21^. Interestingly, our recent transcriptome analysis of G9a knockdown myoblasts^34^ revealed differential expression of several genes that encode sarcomere proteins, including thin actin filament (Actc1), thick myosin filament (Myh3, Myl4), Z-disc components (Actn2, Csrp3, Fbxo32, Myoz2, Unc45b), M-band (Lmod3, Myom2, Myom3), troponin complex (Tnnc2, Tnni2, Tnnt3), titin (Ttn) and melusin (Itgb1bp2) (Fig. 1a). Since MEF2 directly regulates the expression of many sarcomere and structural genes^3^,^20^, we hypothesized that G9a may repress their expression via inhibition of MEF2 transcriptional activity. As a start point, we analyzed the expression of Myom2, Myom3, Actn2, Myoz2 and Ttn in C2C12 cells transfected with small interfering RNA (siRNA) specific for G9a (siG9a) compared to control cells (siControl) transfected with scrambled siRNA. Consistent with the microarray data^34^, the expression of these genes was up-regulated at both mRNA and protein levels in proliferating siG9a cells compared to control cells (Fig. 1b). Similar results were obtained in siG9a primary myoblasts (Supplementary Fig. 1a). To determine if methyltransferase activity is important for repression of these genes, C2C12 cells were treated with UNC0638, a small molecule compound that inhibits G9a activity^35^. Upon treatment with UNC0638, expression of sarcomere genes was increased in C2C12 cells and primary myoblasts (Fig. 1c, Supplementary Fig. 1b), whereas global H3K9me2 was reduced, indicating that the methyltransferase activity of G9a is important for repression of sarcomeric genes. Notably, knock-down of GLP did not result in an increase in sarcomeric genes (Supplementary Fig. 2), suggesting that the increase in sarcomeric gene expression upon UNC0638 treatment is likely due to inhibition of G9a activity. To examine the functional consequence of G9a-mediated regulation of the contractile apparatus, we retrovirally over-expressed G9a (pBABE-G9a) in C2C12 cells (Fig. 1d). The expression of sarcomeric genes was repressed in proliferating G9a over-expressing cells compared to control cells (Fig. 1d). More importantly, upon differentiation, regularly spaced Actn2 staining in the Z-disc, and F-actin in the thin filament, were evident in control cells. In contrast, pBABE-G9a myotubes showed reduced and irregularly spaced Actn2 staining, and F-actin staining did not reveal any discernible structures (Fig. 1e). Together, these results indicate that sarcomeric structure and myofibril formation was perturbed by G9a over-expression.

### G9a deposits repressive epigenetic marks at sarcomeric gene promoters

**Figure 2:**
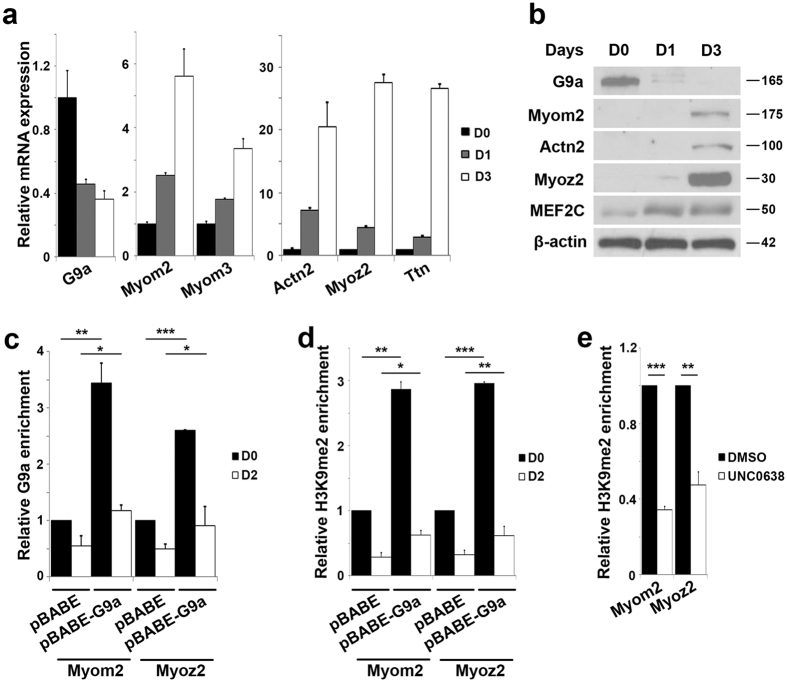
G9a occupancy and H3K9me2 enrichment at sarcomeric gene promoters. (**a**,**b**) Endogenous expression of G9a and sarcomeric genes was analyzed at mRNA (**a**) and protein (**b**) level in myoblasts cultured in growth medium (D0), or one day (D1) and three days (D3) after induction of differentiation. (**c**) G9a occupancy and (**d**) H3K9me2 enrichment at the Myom2 and Myoz2 promoters were examined by ChIP assays in pBABE and pBABE-G9a cells at D0 and two days after differentiation (D2). Enrichment in pBABE-G9a cells was plotted relative to D0 control (pBABE). (**e**) H3K9me2 was examined by ChIP assays at Myom2 and Myoz2 promoters in undifferentiated C2C12 cells treated with DMSO and UNC0638 for 48 hr. Enrichment was normalized to control (DMSO). Error bars indicate mean ± standard error of n = 3.

To examine the mechanisms by which G9a regulates sarcomeric gene expression, we first analyzed the endogenous expression profile of G9a and sarcomeric genes during myogenic differentiation. Consistent with previous studies^32^,^33^,^34^, G9a expression decreased at the mRNA and protein level upon myogenic differentiation and was inversely correlated with expression of sarcomere genes and MEF2C (Fig. 2a,b).

G9a primarily represses its target genes through mediation of histone H3 lysine 9 di-methylation (H3K9me2) repression marks. We therefore performed chromatin immunoprecipitation (ChIP) assays for G9a occupancy and H3K9me2 enrichment at the Myom2 and Myoz2 promoters which have been previously characterized as MEF2 targets^20^,^36^. In control cells, G9a occupancy (Fig. 2c) and its signature H3K9me2 enrichment (Fig. 2d) were apparent at these promoters in undifferentiated myoblasts (D0), and the occupancy was reduced during differentiation (D2). In pBABE-G9a cells, G9a occupancy and H3K9me2 marks were increased both at the undifferentiated state and upon differentiation. Moreover, this repression mark present at both promoters was reduced in UNC0638-treated cells (Fig. 2e) indicating that G9a represses expression of these genes in a methyltransferase activity-dependent manner.

### G9a interacts with and inhibits MEF2C activity

**Figure 3:**
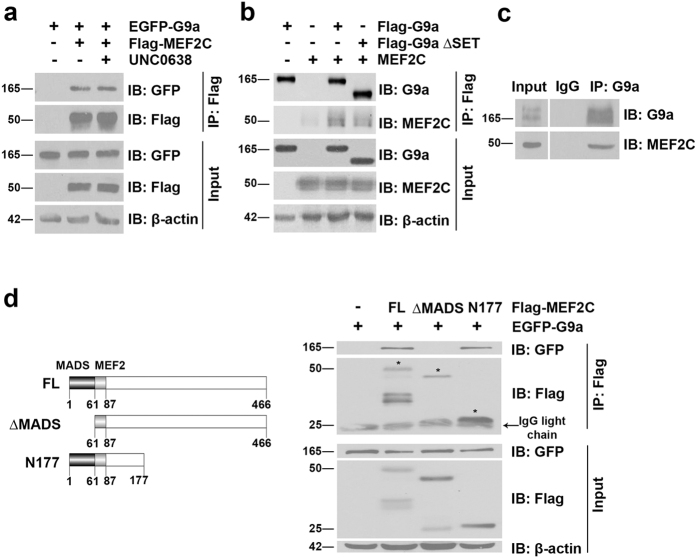
G9a interacts with MEF2C. (**a**) Flag-MEF2C and EGFP-G9a were transfected in HEK293T cells which were subsequently treated with UNC0638 for 48 hr. MEF2C was immunoprecipitated with anti-Flag agarose beads and immunoblotted with anti-Flag and anti-GFP antibodies. 10% lysates (Input) were probed for G9a and MEF2C expression. β-actin was used as a loading control. (**b**) MEF2C was co-transfected with Flag-G9a or Flag-G9a ∆SET in HEK293T cells. G9a was immunoprecipitated using anti-Flag agarose beads and probed for association with MEF2C. 10% lysates were analyzed for G9a and MEF2C expression. (**c**) Endogenous G9a was immunoprecipitated from C2C12 lysates and probed for association with endogenous MEF2C. IgG was used as a negative control. Input shows expression of G9a and MEF2C in the lysates. (**d**) Schematic representation of full length MEF2C (FL), MEF2C lacking the MADS domain (∆MADS), and MEF2C with a truncated C-terminus (N177). Numbers indicate amino acid residues on MEF2C. EGFP-G9a was co-expressed with FL, ∆MADS, and N177. MEF2C was immunoprecipitated with anti-Flag agarose beads and lysates were probed for association with G9a using anti-GFP antibody. The expression of full length and MEF2C deletion mutants was analyzed in immunoprecipitates with anti-Flag antibody. Input refers to G9a and MEF2C expression in the lysates. β-actin was used as a loading control. Asterix indicates specific MEF2C bands.

While all MEF2 factors are expressed in skeletal muscle, only the knock-out of MEF2C in mice disrupts sarcomere organization^20^. We therefore examined whether G9a-mediated repression of sarcomeric genes was mediated through an impact on MEF2C. Due to high transfection efficiency, we first checked whether G9a interacts with MEF2C in HEK293T cells. EGFP-G9a and Flag-MEF2C were expressed individually or together. Immunoprecipitation of MEF2C revealed a clear interaction with G9a that was not altered upon UNC0638 treatment (Fig. 3a). In reciprocal experiments, MEF2C was co-immunoprecipitated with full length G9a as well as G9a lacking the SET domain (G9a ∆SET) (Fig. 3b), suggesting that the SET domain is not essential for interaction with MEF2C. To confirm their association in myogenic cells, we immunoprecipitated endogenous G9a from myoblasts and verified its association with endogenous MEF2C (Fig. 3c). To identify the domain in MEF2C that interacts with G9a, Flag-MEF2C deletion mutants (Fig. 3d, left panel) were over-expressed together with EGFP-G9a. G9a immunoprecipitated with full-length MEF2C (FL) and with MEF2C lacking the C-terminal transactivation region (N177), but not with a MEF2C deletion mutant lacking the MADS domain (∆MADS) (Fig. 3d, right panel).

**Figure 4:**
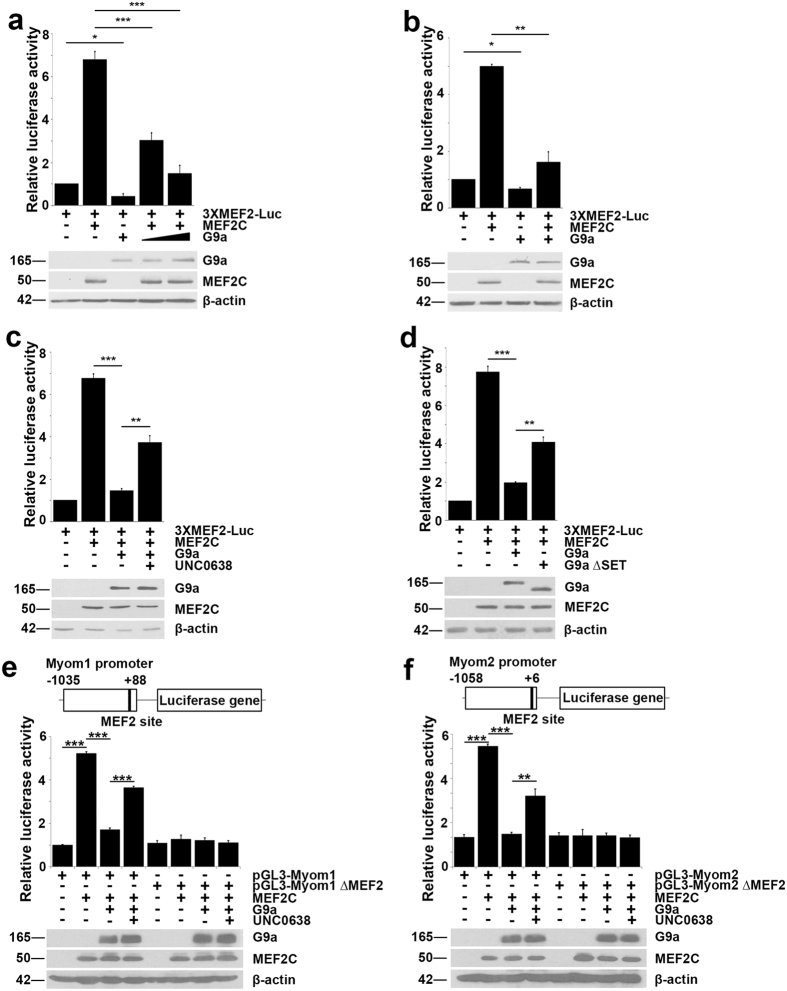
G9a represses MEF2C transcriptional activity. (**a**,**b**) Luciferase assays were performed using 100ng of 3XMEF2-Luc reporter in (**a**) 10T1/2 fibroblasts or (**b**) C2C12 myoblasts co-transfected with 100 ng Flag-MEF2C in the presence or absence of 50 and 100 ng Flag-G9a as indicated. Luciferase activity was measured 24 hr after transfection. Lysates were analyzed by western blot for exogenous G9a and MEF2C expression. (**c**) 100 ng 3XMEF2-Luc reporter was transfected in 10T1/2 fibroblasts with 100 ng Flag-MEF2C and 100 ng Flag-G9a. Cells were recovered for 24 hr after transfection and then treated with UNC0638 for 48 hr, and luciferase activity was measured after treatment. (**d**) 100 ng 3XMEF2-Luc reporter was transfected with 100 ng Flag-MEF2C and 100 ng Flag-G9a or 200 ng Flag-G9a∆SET. Luciferase activity was measured 24 hr after transfection. Western blot was done to analyze expression of G9a and MEF2C in the lysates. Error bars indicate mean ± standard error of n = 3. (**e**,**f**) Schematic diagram depicting Myom1 (pGL3-Myom1) and Myom2 (pGL3-Myom2) promoter luciferase constructs. The MEF2 binding site within the promoters is indicated and was mutated (pGL3-Myom1∆MEF2 and pGL3-Myom2∆MEF2 respectively). Luciferase assays were done using pGL3-Myom1 and pGL3-Myom1∆MEF2 (**e**), and pGL3-Myom2 and pGL3-Myom2∆MEF2 (**f**) in 10T1/2 fibroblasts co-transfected with MEF2C in the presence or absence of G9a, treated with UNC0638 or DMSO for 48 hr. Western blot shows expression of exogenous G9a and MEF2C. Error bars indicate mean ± standard deviation. The data are representative of three independent experiments.

We then examined the impact of G9a on MEF2 transcriptional activity. C3H10T1/2 (10T1/2) fibroblasts were transfected with a synthetic MEF2-responsive reporter (3XMEF2-Luc) that contains three MEF2-binding sites upstream of the luciferase gene^9^. As expected, the reporter was activated in response to MEF2C. G9a inhibited MEF2C activity in a dose-dependent manner in 10T1/2 cells (Fig. 4a) as well as in C2C12 myoblasts (Fig. 4b). As G9a-mediated regulation of sarcomeric genes involves its methyltransferase activity, we tested if repression of MEF2C transcriptional activity is dependent on its catalytic activity. Treatment of cells with UNC0638 rescued G9a-mediated repression of MEF2C (Fig. 4c). Consistently, repression of MEF2C was significantly lesser in the presence of G9a ∆SET compared to full length G9a (Fig. 4d). To validate these findings on natural MEF2 target gene promoters, we analyzed Myom1 and Myom2 that have previously been characterized as direct MEF2C targets^20^. In the presence of G9a, MEF2C-mediated activation of both promoters was inhibited (Fig. 4e,f). Moreover, mutation of MEF2 DNA-binding site in both promoters (pGL3-Myom1∆MEF2 and pGL3-Myom2∆MEF2 respectively) abrogated the impact of MEF2C and G9a, and UNC0638 treatment de-repressed MEF2C activity.

### MEF2C over-expression rescues sarcomere perturbations in G9a over-expressing cells

**Figure 5:**
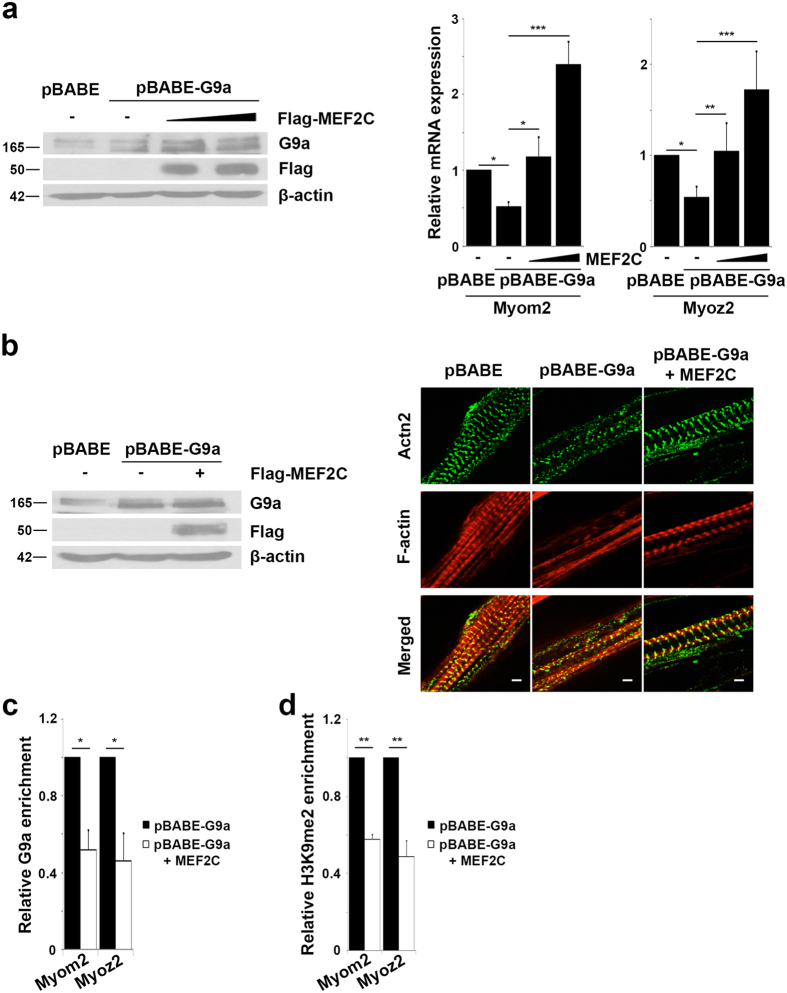
MEF2C over-expression rescues G9a-mediated sarcomere perturbations. (**a**) 1.5 and 3 μg of Flag-MEF2C was transfected in pBABE-G9a myoblasts. Expression of G9a, and MEF2C was analyzed by western blot. Myom2 and Myoz2 expression was analyzed in proliferating cells by qPCR. (**b**) 0.5 μg Flag-MEF2C was transfected in pBABE-G9a cells. The expression of MEF2C and G9a was analyzed by western blot (left panel). pBABE, pBABE-G9a and pBABE-G9a expressing Flag-MEF2C were differentiated for six days and stained for Actn2 and F-actin (right panel). Confocal images were taken at 100X magnification with 3X focus. Scale bar: 5 μm. (**c**,**d**) pBABE-G9a cells transfected with empty vector or MEF2C were analyzed by ChIP assays for G9a occupancy (**c**) and H3K9me2 enrichment (**d**). Enrichment at promoters was analyzed by qPCR and normalized to pBABE-G9a cells transfected with empty vector. Error bars indicate mean ± standard error of n = 3.

Given the repression of MEF2C transcriptional activity, we tested if repression of sarcomeric genes and sarcomere organization by G9a is MEF2C-dependent. Expression of exogenous MEF2C in pBABE-G9a cells (Fig. 5a, left panel) rescued Myom2 and Myoz2 expression in a dose-dependent manner (Fig. 5a, right panel). Moreover, perturbations of sarcomere assembly in G9a over-expressing cells as seen by Actn2 and F-actin staining were rescued by exogenous MEF2C expression (Fig. 5b). Correspondingly, G9a occupancy and H3K9me2 marks were reduced at Myom2 and Myoz2 promoters in G9a over-expressing cells transfected with MEF2C (Fig. 5c,d).

### G9a promotes HDAC5 association with MEF2C

**Figure 6:**
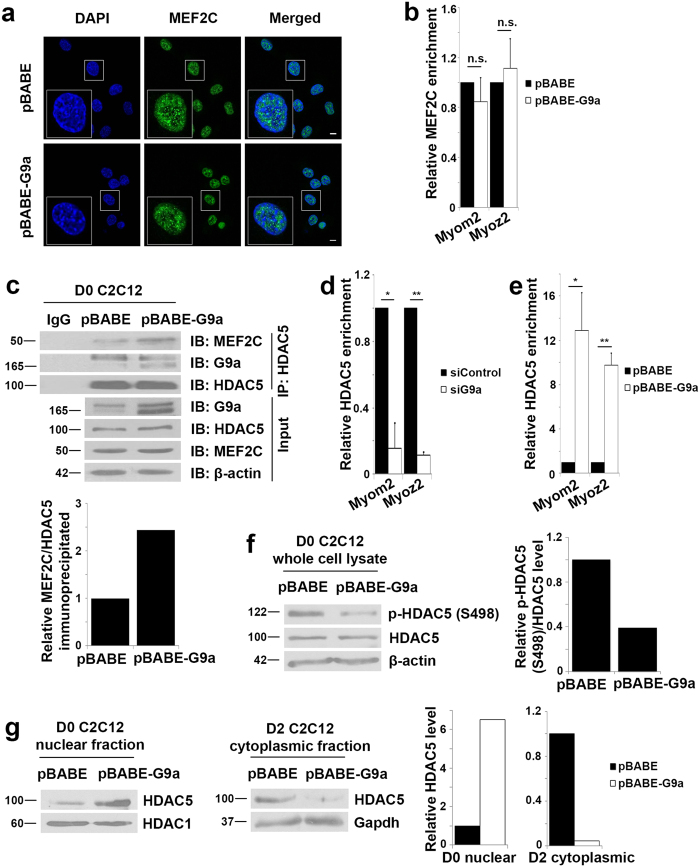
G9a regulates MEF2C-HDAC5 association. (**a**) Sub-cellular localization of MEF2C (green) was examined in proliferating pBABE and pBABE-G9a C2C12 cells by confocal microscopy. Nuclei were stained with DAPI. Images were taken at 100X magnification. Scale bar: 10 μm. (**b**) MEF2C binding at the Myom2 and Myoz2 promoters was analyzed by ChIP assays in undifferentiated control and pBABE-G9a cells. Enrichment promoters was probed for by qPCR and normalized to control (pBABE). n.s. *p*-value not significant. (**c**) Endogenous HDAC5 was immunoprecipitated from lysates of proliferating pBABE and pBABE-G9a cells. Immunoprecipitates were analyzed with anti-G9a, anti-MEF2C and anti-HDAC5 antibodies. Input shows expression of G9a, HDAC5 and MEF2C expression in lysates. The lower panel shows densitometric analysis of the western blot. (**d**,**e**) HDAC5 occupancy at Myom2 and Myoz2 promoters was probed by ChIP in undifferentiated siControl and siG9a cells (**d**) and pBABE and pBABE-G9a cells (**e**). (**f**) Whole cell lysates from undifferentiated pBABE and pBABE-G9a cells were probed for p-HDAC5 (S498). Densitometric analysis of p-HDAC5 (S498)/HDAC5 levels is shown in the right panel. (**g**) Nuclear fractions from undifferentiated (D0) and cytoplasmic fractions from differentiated (D2) pBABE and pBABE-G9a cells were probed for HDAC5. HDAC1 and Gapdh served as loading controls for nuclear and cytoplasmic fractions respectively. Densitometric analysis of the western blots showed increased nuclear HDAC5 and reduced cytoplasmic HDAC5 in pBABE-G9a lysates.

To examine the mechanisms by which G9a inhibits MEF2C transcriptional activity, we analyzed MEF2C sub-cellular localization. MEF2C was found solely in the nucleus, and no difference was observed between pBABE control cells and pBABE-G9a cells (Fig. 6a). In addition, no changes in MEF2C occupancy at Myom2 and Myoz2 promoters were apparent (Fig. 6b) indicating G9a does not alter MEF2C localization or its DNA-binding ability.

**Figure 7:**
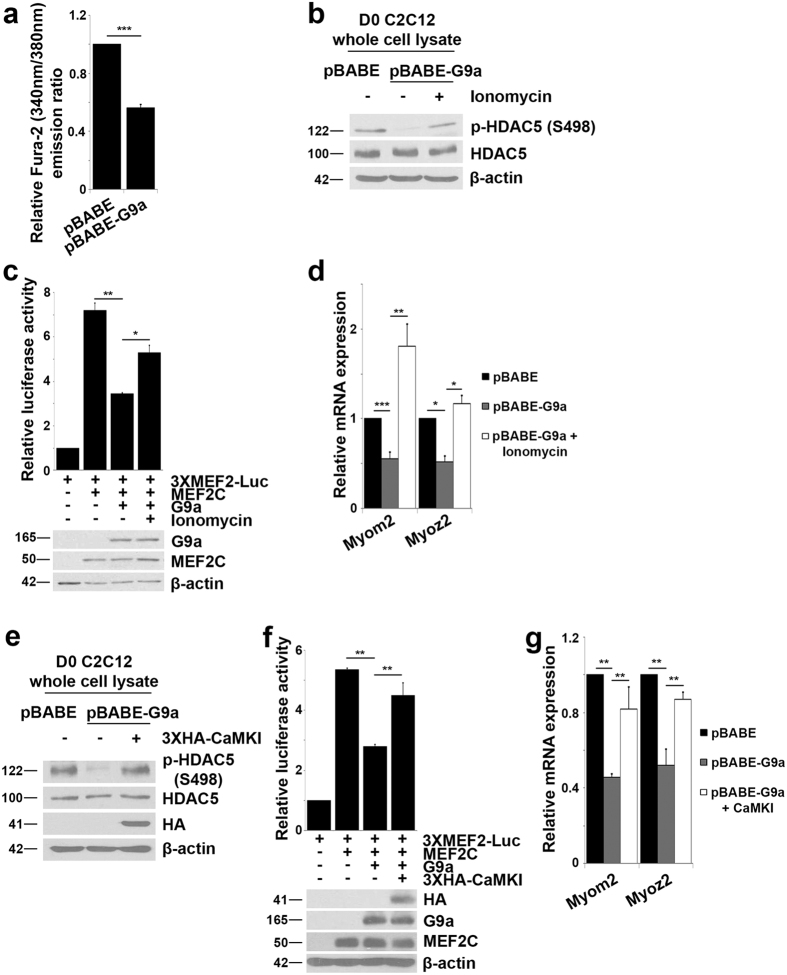
Calcium signaling rescues G9a-mediated repression of MEF2C activity. (**a**) Intracellular calcium was detected in pBABE and pBABE-G9a myoblasts by incubation with Fura-2 AM. Fura-2 fluorescence at 340/380 nm in pBABE-G9a cells is shown relative to control cells. (**b**) Whole cell lysates from pBABE-G9a myoblasts treated with 2 μM of ionomycin were analyzed for levels of p-HDAC5 (S498). (**c**) 100 ng of 3XMEF2-Luc reporter was transfected in C2C12 myoblasts along with 100 ng Flag-MEF2C, and 50 ng Flag-G9a as indicated. Cells were treated with 2 μM of ionomycin or DMSO as control for 4 hr and luciferase assays were performed. Lysates were analyzed for G9a and MEF2C expression by western blot. (**d**) Expression of Myom2 and Myoz2 were analyzed by qPCR in proliferating pBABE-G9a cells treated with 2 μM of ionomycin for 4 hr. (**e**) Constitutively active CaMKI was transfected in pBABE-G9a myoblasts. Whole cell lysates were analyzed for levels of p-HDAC5 (S498). (**f**) Luciferase assay was performed on C2C12 myoblasts transfected with 100 ng of 3X-MEF2-Luc reporter along with 100 ng Flag-MEF2C, 100ng Flag-G9a and 50 ng 3XHA-CaMKI as indicated. Lysates were analyzed for CaMKI, G9a and MEF2C expression by western blot. (**g**) Expression of Myom2 and Myoz2 were analyzed by qPCR in pBABE-G9a myoblasts transfected with 3XHA-CaMKI. Error bars indicate mean ± standard error of n = 3.

Class II HDACs are well-known repressors of MEF2 activity^6^. Notably, class II HDACs have been also implicated in regulation of sarcomeric gene expression by repressing MEF2 activity^22^,^24^. We therefore examined if G9a modulates this regulatory axis. We focused on HDAC5 because HDAC4 is predominantly cytoplasmic in undifferentiated C2C12 cells^37^. To examine whether G9a has an impact on the association of MEF2 and HDAC5, HDAC5 was immunoprecipitated in control and G9a over-expressing cells. As expected, interaction of HDAC5 and MEF2C was apparent in undifferentiated control cells. Interestingly, this association was enhanced in pBABE-G9a cells despite MEF2C and HDAC5 being expressed at the same level as control cells (Fig. 6c). Moreover, HDAC5 occupancy at Myom2 and Myoz2 gene promoters was decreased in siG9a myoblasts (Fig. 6d) and increased in pBABE-G9a myoblasts (Fig. 6e). During myogenic differentiation, in response to calcium signaling, HDAC5 is phosphorylated at serine (S)259 and S498 and shuttled to the cytoplasm^38^. Given the enhanced association of HDAC5 with MEF2C in proliferating G9a over-expressing cells, we wondered whether phosphorylation and subsequent dissociation of HDAC5 from MEF2C was affected. The level of p-HDAC5 (S498) was reduced in G9a over-expressing cells compared to control cells (Fig. 6f). We then examined HDAC5 localization in fractioned nuclear and cytoplasmic lysates from control and G9a over-expressing cells. In undifferentiated G9a over-expressing cells, nuclear HDAC5 levels were increased compared to control cells. Correspondingly, the levels were reduced in the cytoplasm in differentiated cells (Fig. 6g). Together, these results suggest that G9a may inhibit calcium signaling and thereby regulate the association of MEF2C with HDAC5. To test this directly, we used a calcium-selective indicator Fura-2 AM. Intracellular Ca^2+^ levels were lower in undifferentiated pBABE-G9a cells relative to control cells (Fig. 7a). We then examined HDAC5 phosphorylation and the expression of MEF2-dependent sarcomeric genes in cells treated with ionomycin, which promotes influx of extracellular calcium into cells to trigger calcium signaling. In response to ionomycin, increased HDAC5 phosphorylation at S498 was apparent in G9a over-expressing cells (Fig. 7b). Concomitantly, a rescue of MEF2C transcriptional activity (Fig. 7c) and expression of MEF2C target genes Myom2 and Myoz2 was evident (Fig. 7d). Similarly, when constitutively active CaMKI was expressed in pBABE-G9a cells, a higher level of HDAC5 phosphorylation (Fig. 7e) coincided with increased MEF2C activity (Fig. 7f) and MEF2C target gene expression (Fig. 7g).

## Discussion

The results of this study reveal for the first time that G9a epigenetically controls structural elements in skeletal muscle. G9a interacts with the MEF2C-HDAC5 complex in proliferating myoblasts, and mediates repressive H3K9me2 marks at MEF2 sites in sarcomeric gene promoters that lead to disrupted sarcomere organization.

G9a is expressed in proliferating myoblasts and orchestrates repression of skeletal myogenesis at multiple levels. In addition to regulating cell cycle progression^34^ and the expression of differentiation specific genes^32^,^33^,^39^, G9a represses maturation by inhibiting expression of sarcomere genes in proliferating myoblasts. The expression of sarcomere/structural genes is up-regulated in proliferating G9a knock down cells. Moreover, G9a occupancy and H3K9me2 enrichment are apparent at sarcomere gene promoters in growth conditions. These observations argue that repression of sarcomere genes is not secondary to G9a-mediated block of myogenic differentiation, but rather reflect an active transcriptional control of contractile apparatus. Consistent with this contention, conditional ablation of G9a in the postnatal brain using *Camk2a*-Cre results in the up regulation of muscle structural and sarcomere genes in neurons^31^ validating that G9a represses muscle sarcomere and structural genes *in vivo*, independent of myogenic differentiation.

Among the various transcription factors that regulate expression of sarcomere genes in skeletal muscle, MEF2 factors are critical^40^. Disruption of MEF2C has been shown to perturb sarcomere morphology and integrity in various species due to reduced expression of M-line and Z-disc genes. G9a does not appear to inhibit MEF2C expression or block MEF2C DNA-binding ability. However, G9a interacts with MEF2C and blocks its transcriptional activity by enhancing its association with HDAC5. Class II HDACs associate with HDAC3/SMRT/NCoR complex^7^, as well as SUV39H1/HP1 to mediate transcriptional repression^8^. G9a has also been shown to interact with class I HDACs^41^, as well as with SUV39H1 and SETDB1^42^. Thus G9a is likely present in multi-protein repressor complexes that assemble to block MEF2 function in proliferating myoblasts. Class II HDACs are known to undergo nucleocytoplasmic shuttling in response to various signals. During myogenic differentiation, CaMK phosphorylates HDAC5 at S259 and S498 that leads to recruitment of 14-3-3 proteins and its nuclear export. In addition, cAMP/PKA also phosphorylates HDAC5 at S280 to block its nuclear export^43^. A reduction in p-HDAC5 S498 is apparent in G9a overexpressing cells. Conversely, activation of calcium signaling or expression of constitutively active CaMK rescues p-HDAC5 S498 levels and sarcomere gene expression in presence of G9a. Together, these data indicate that G9a modulates calcium signaling dependent HDAC5 dissociation from MEF2C, rather than impact PKA-mediated phosphorylation of HDAC5. Consistent with this notion, nuclear HDAC5 is increased in undifferentiated G9a over-expressing cells, along with a decrease in the cytoplasmic pool in differentiated cells. Nuclear retention of HDAC5 in G9a overexpressing cells may account for the increased association of MEF2 and HDAC5.

In addition to sarcomere genes, MEF2 and class II HDACs (HDAC4) regulate many components of the costamere. Moreover, dystrophin, β- and γ-sarcoglycan of the dystrophin-glycoprotein complex (DGC) are also targets of the class II HDAC-MEF2 axis^24^,^44^. Interestingly, the expression of β and γ sarcoglycans were also up-regulated in G9a knockdown myoblasts (not shown), suggesting that G9a may also directly regulate components of the DGC complex and the costamere genes in skeletal muscle. The impact of G9a in the regulation of muscle contractile machinery may not be mediated exclusively through MEF2C. G9a influences the function of number of transcription factors such as MyoD^32^, HIF1α^45^, NF-kB^46^ that play roles in sarcomere and structural gene expression^40^.

In line with our observations, G9a was recently shown to repress Myh6, an isoform of myosin heavy chain, that is expressed in the heart and whose loss of expression causes contractile dysfunction. G9a expression is elevated in human hypertrophic hearts and contributes to hypertrophic cardiomyopathy^47^. It is noteworthy that hypertrophic cardiomyopathy is found in patients with mutations in MYOM1^48^, ACTN2^49^ and MYOZ2^50^, and deletion of Myoz2 in mice increases susceptibility to hypertrophic cardiomyopathy upon cardiac stress^51^. On the other hand, G9a expression is decreased in mouse models of dilated cardiomyopathy^52^ whereas increased MEF2 activity in the heart causes dilated cardiomyopathy^53^,^54^. The impact of G9a on the MEF2-class II HDACs regulatory axis may be relevant in cardiac myopathies associated with dysregulation of sarcomere genes. Pharmacological targeting of G9a activity may be a viable therapeutic avenue in skeletal and cardiac pathologies associated with dysregulation of the MEF2-class II HDAC regulatory circuit.

## Materials and Methods

### Cell culture

C2C12 mouse myoblasts were cultured in growth medium [Dulbecco’s Modified Eagle’s Medium (DMEM) high glucose supplemented with 20% fetal bovine serum (FBS)]. To induce differentiation, cells were cultured in differentiation medium (DMEM supplemented with 2% horse serum). Primary myoblast cultures were established from C57BL/6 wild type mice as previously described^55^. All experiments were done in accordance to relevant guidelines. The experimental protocols were approved by the National University of Singapore Institutional Animal Care and Use Committee. Purity of myoblasts was checked by Pax7 immunostaining, and cells were used only when Pax7 positivity was >95%. Primary myoblasts were cultured on collagen-coated plates in primary culture medium [Ham’s F-10 supplemented with 20% FBS and 5ng/ml basic fibroblast growth factor (bFGF)]. HEK293T, Phoenix and C3H10T1/2 (10T1/2) cells were grown in DMEM supplemented with 10% FBS. All cells were incubated at 37°C with 5% CO_2_. For inhibition of methyltransferase activity, cells were cultured in growth medium containing 250 nM of UNC0638^35^ for 48 hours (hr) before being harvested. For induction of calcium uptake, C2C12 cells were treated with 2 μM of ionomycin in growth medium for 4 hr.

### Plasmids

pBABE-G9a, pcDNA2-Flag-G9a and pcDNA2-Flag-G9a ∆SET, pEGFP-G9a^56^, and pcDNA3-Flag-HDAC5 were gifts from Dr. Martin Walsh (Mount Sinai School of Medicine, New York, NY, USA), 3XMEF2-Luc was obtained from Dr. Andrew Lassar (Harvard Medical School, Boston, MA, USA), pFlag-CMV2-FL MEF2C, ∆MADS and N177 were provided by Dr. Lizi Wu (University of Florida, Gainesville, FL, USA)^57^ and pCMX-Flag-MEF2C was provided by Dr. Ronald Evans (Salk Institute for Biological Sciences, La Jolla, CA, USA). pcDNA3.1-3X-HA-CaMKI (N-term) was from Dr. Eric Olson (University of Texas Southwestern Medical Center, Dallas, TX, USA)^38^. Mouse Myom1 promoter (−1035 to +88) and Myom2 promoter (−1058 to +6) were cloned into pGL3-basic vector (Promega) using primers described previously^20^. Amplification of the Myom1 and Myom2 promoters were performed using the Expand High Fidelity PLUS PCR System (Roche), with mouse chromatin from C2C12 cells as template. Amplicons were digested with KpnI and HindIII for Myom1 promoter, or MluI and HindIII for Myom2 promoter (New England Biolabs), for which restriction sites were introduced into the PCR primers, and ligated into the corresponding sites on the pGL3-basic vector to produce pGL3-Myom1 and pGL3-Myom2 reporter constructs. For pGL3-Myom1 ∆MEF2 and pGL3-Myom2 ∆MEF2, four residues within each of the two MEF2 binding sites were mutated that were shown to abrogate MEF2 binding^20^. The point mutations were introduced using the QuikChange Site-Directed Mutagenesis Kit (Stratagene) according to manufacturer’s instructions. Primers used for mutagenesis are:

pGL3-Myom1∆MEF2:5′-CCCCTCCCCATGTGCTGCTACCGGTATCTGCCTTCCTGGCCATA-3′ (forward) and 5′-TATGGCCAGGAAGGCAGATACCGGTAGCAGCACATGGGGAGGGG-3′ (reverse);

pGL3-Myom2∆MEF2:5′-GCCAGAGGAGGTGCTACCGGTAGGCAAGGGACTGCCTCTCC-3′ (forward) and 5′-GGAGAGGCAGTCCCTTGCCTACCGGTAGCACCTCCTCTGGC-3′ (reverse).

Underlined residues indicate sites of mutation. Constructs were sequenced to confirm the presence of the mutations.

### siRNA and retroviral infections

100 nM siRNA specific for G9a (siG9a; L-053728-01, ON-TARGETplus SMARTpool, Dharmacon), 50 nM siRNA specific for GLP (siGLP; L-059041-01, ON-TARGETplus SMARTpool, Dharmacon) or control scrambled siRNA (siControl; D-001810-10 ON-TARGETplus Non-Targeting Pool, Dharmacon) were transfected in proliferating C2C12 cells or primary myoblasts using Lipofectamine RNAiMAX (Invitrogen). Cells were harvested 48 hr post transfection. To over-express G9a in C2C12 cells, retroviral vectors pBABE-G9a or empty vector (pBABE) were transfected into Phoenix packaging cells using Calcium Phosphate Transfection Kit (Invitrogen). Medium was harvested after 24 hr and filtered with 0.45 μm syringe filter. For transduction of C2C12 myoblasts, polybrene was added to a final concentration of 8 μg/ml. Selection was performed with growth medium containing 2 μg/ml of puromycin for 48 hr.

### RNA isolation and quantitative real-time polymerase chain reaction (qPCR)

Total RNA from cells was extracted using TRIzol reagent (Invitrogen) and treated with DNase using the TURBO DNA-free Kit (Life Technologies). For qPCR, 1 μg of RNA was converted to complementary DNA (cDNA) with the Superscript III First-Strand Synthesis System using oligo-dT primers (Invitrogen) and subsequently treated with RNase H. The cDNA was then probed through qPCR in triplicates using the LightCycler 480 SYBR Green I Master mix (Roche) and 0.2 μM of primers and detected using the LightCycler 480 I instrument (Roche) with the following PCR parameters: denaturation at 95 °C for 5 min; amplification for 45 cycles of 95°C for 10 s, 60°C for 10 s and 72°C for 10 s. Melting curve analysis was tested to ensure presence of only a single amplicon product for each primer. Threshold cycle (Ct) values were analyzed on default settings. Relative mRNA expression was calculated using 2^−∆Ct^ method upon normalization to Ct for Gapdh and expressed as fold change relative to the respective control. Primer sequences for G9a and Gapdh are as previously published^32^. Primers for Myom2, Myom3, Myoz2, Actn2 and Ttn were validated and obtained from Primer Bank^58^.

### Immunoprecipitation (IP) assay, fractionation, and immunoblotting

Protein lysates were harvested from cells in Radioimmunoprecipitation assay (RIPA) lysis buffer [50 mM NaCl, 50 mM Tris-HCl pH 6.8, 1 mM EDTA, 1% NP-40, 0.1% sodium deoxycholate, protease inhibitor cocktail (Roche)]. Histones were isolated using RIPA lysis buffer supplemented with 2% sodium dodecyl sulphate (SDS).

For cytoplasmic and nuclear fractionation, cells were re-suspended in cytoplasmic extraction buffer [10 mM HEPES, pH7.5, 10 mM KCl, 0.1 mM EDTA, 0.5 mM DTT, 0.1% NP-40 and Protease and Phosphatase Inhibitor (Thermo Scientific)] and incubated on ice for 15 minutes. Lysates were centrifuged at 12,000 g for 10 minutes and supernatant was harvested as cytoplasmic extract. The pellets were washed thrice in cytoplasmic extraction buffer without NP-40. After the last wash, the pellet was re-suspended in RIPA lysis buffer and harvested as nuclear extract.

For immunoprecipitation, lysates were incubated with anti-Flag agarose beads (Sigma-Aldrich). To immunoprecipitate endogenous G9a and HDAC5, 1 mg of lysate was incubated with 2 μg of primary antibody [anti-G9a (ab40542, Abcam), anti-HDAC5 (40970, Active Motif), or normal rabbit IgG (sc-2027, Santa Cruz Biotechnology)]. Primary antibodies for immunoblotting include anti-β-actin (A1978, Sigma-Aldrich), anti-Flag (F3165, Sigma-Aldrich), anti-G9a (3306S, Cell Signaling), anti-GLP (ab41969, Abcam) anti-GFP (sc-9996, Santa Cruz Biotechnology), anti-MEF2C (sc-13266, Santa Cruz Biotechnology), anti-HDAC5 (40970, Active Motif), anti-p-HDAC5 (S498) (ab47283, Abcam), anti-HDAC1 (#05–100, Millipore), anti-Gapdh (sc-25778, Santa Cruz Biotechnology), anti-Actn2 (sc-17829, Santa Cruz Biotechnology), anti-Myom2 (ab93915, Abcam), anti-Myoz2 (11450-1-AP, ProteinTech), anti-H3K9me2 (#9753, Cell Signaling), and anti-H3 (ab1791, Abcam).

### Luciferase assays

Luciferase assays were performed using the Dual Luciferase Reporter Assay kit (Promega). Briefly, cells were transfected with 100 ng of 3XMEF2-Luc or pGL3-Myom1 or pGL3-Myom2 reporter together with 3 ng of Renilla reporter in the presence or absence of 100 ng of Flag-MEF2C and 50 or 100 ng of Flag-G9a, or 200 ng of Flag-G9a∆SET. After 24 hr, cells were washed with PBS and lysed with Passive Lysis Buffer. Readings were taken using the Varioskan Flash Multimode Reader (Thermo Scientific). Where indicated, 24 hr after transfection, cells were treated with 250 nM UNC0638 for 48 hr. For ionomycin treatment, cells were allowed to recover after transfection for 24 hr before being treated for 4 hr with 2 μM of ionomycin.

### Chromatin immunoprecipitation (ChIP)

ChIP was performed as previously described^32^ using the ChIP Assay Kit (Millipore) using 1 million C2C12 cells and 2 μg of anti-G9a (ab40542, Abcam), anti-H3K9me2 (#17–681, Millipore), anti-HDAC5 (40970, Active Motif) and anti-MEF2C (ab79436, Abcam) antibodies. DNA was isolated using phenol-chloroform. To enhance yield of DNA, 100% ethanol and glycogen were added and incubated overnight at −20 °C. Enrichment of Myom2 and Myoz2 promoter regions was probed in triplicates using qPCR. Relative enrichment was calculated using 2^−∆Ct^ method, normalizing to DNA isolated from 10% input. Primers used for ChIP-qPCR are: Myom2 promoter 5′-ATTGCTCCCCTTGCATCTGG-3′ (forward) and 5′- ACACTGGCCCAAGACAGCAG-3′ (reverse); Myoz2 promoter 5′- TCTTCAGGCCCAGTGAATGA-3′ (forward) and 5′- CAGGGGACTGAGCCTTTCTG-3′ (reverse).

### Immunofluorescence

C2C12 cells were cultured on Thermonax plastic cover slips. Cells were washed with PBS and fixed with 4% paraformaldehyde (PFA). Fixed cells were blocked and permeabilized with 0.1% Triton-X-100 in PBS supplemented with 10% horse serum. After blocking, cells were incubated with primary antibody in PBS with 10% horse serum. The next day, cells were washed with PBS and probed with secondary antibody conjugated to Alexa-Fluor 488 (Invitrogen) or Alexa-Fluor 633 (Invitrogen). Cells were washed with PBS thrice, and counter-stained with DAPI before being viewed under BX53 fluorescence microscope or FluoView FV1000 confocal fluorescence microscope (Olympus). Primary antibodies used for immunofluorescence are anti-Actn2 (A7811, Sigma-Aldrich; 1:500), and anti-MEF2C (sc-13266, Santa Cruz Biotechnology;1:200). Rhodamine-phalloidin (R415, Life Technologies; 1:200) was used for staining filamentous actin (F-actin).

### Intracellular calcium assay

Undifferentiated pBABE and pBABE-G9a cells cultured in flat-bottom black 96-well plates were loaded with 5 μM Fura-2 AM (F1201, Life Technologies) in PBS for 30 min at 37 °C, after which excess Fura-2 AM was washed off. Intracellular levels of Ca^2+^ was then measured using Varioskan Flash Multimode Reader (Thermo Scientific) with excitation wavelength of 340 nm or 380 nm with 5 nm bandwidth, and detection of emission at 510 nm for both excitation wavelengths at 1 s interval. Levels of intracellular Ca^2+^ was calculated as a ratio of emission at 340 nm over emission at 380 nm and shown relative to control.

### Statistical analysis

Error bars indicate mean ± standard error (s.e.m.). Statistical significance was calculated using unpaired two-tailed Student’s T-test of three biological replicates (n = 3), unless otherwise indicated, and *p*-values of <0.05 were considered to be significant. Asterisks indicate different degrees of significance: *p-value <0.05; **p-value <0.01; ***p-value <0.001.

## Additional Information

**How to cite this article**: Ow, J. R. *et al*. G9a inhibits MEF2C activity to control sarcomere assembly. *Sci. Rep*. **6**, 34163; doi: 10.1038/srep34163 (2016).

## Supplementary Material

Supplementary Information

## References

[b1] Molkentin J. D., Black B. L., Martin J. F. & Olson E. N. Cooperative activation of muscle gene expression by MEF2 and myogenic bHLH proteins. Cell 83, 1125–1136 (1995). 8548800 10.1016/0092-8674(95)90139-6

[b2] Kaushal S., Schneider J. W., Nadal-Ginard B. & Mahdavi V. Activation of the myogenic lineage by MEF2A, a factor that induces and cooperates with MyoD. Science 266, 1236–1240 (1994). 7973707 10.1126/science.7973707

[b3] Black B. L. & Olson E. N. Transcriptional control of muscle development by myocyte enhancer factor-2 (MEF2) proteins. Annu. Rev. Cell Dev. Biol. 14, 167–196 (1998). 9891782 10.1146/annurev.cellbio.14.1.167

[b4] Shore P. & Sharrocks A. D. The MADS-box family of transcription factors. Eur. J. Biochem. 229, 1–13 (1995). 7744019 10.1111/j.1432-1033.1995.tb20430.x

[b5] Molkentin J. D., Black B. L., Martin J. F. & Olson E. N. Mutational analysis of the DNA binding, dimerization, and transcriptional activation domains of MEF2C. Mol. Cell. Biol. 16, 2627–2636 (1996). 8649370 10.1128/mcb.16.6.2627 PMC231253

[b6] Lu J., McKinsey T. A., Zhang C. L. & Olson E. N. Regulation of skeletal myogenesis by association of the MEF2 transcription factor with class II histone deacetylases. Mol. Cell 6, 233–244 (2000). 10983972 10.1016/s1097-2765(00)00025-3

[b7] Fischle W. . Enzymatic activity associated with class II HDACs is dependent on a multiprotein complex containing HDAC3 and SMRT/N-CoR. Mol. Cell 9, 45–57 (2002). 11804585 10.1016/s1097-2765(01)00429-4

[b8] Zhang C. L., McKinsey T. A. & Olson E. N. Association of class II histone deacetylases with heterochromatin protein 1: potential role for histone methylation in control of muscle differentiation. Mol. Cell. Biol. 22, 7302–7312 (2002). 12242305 10.1128/MCB.22.20.7302-7312.2002 PMC139799

[b9] Lu J., McKinsey T. A., Nicol R. L. & Olson E. N. Signal-dependent activation of the MEF2 transcription factor by dissociation from histone deacetylases. Proc. Natl. Acad. Sci. USA 97, 4070–4075 (2000). 10737771 10.1073/pnas.080064097 PMC18151

[b10] McKinsey T. A., Zhang C. L. & Olson E. N. Activation of the myocyte enhancer factor-2 transcription factor by calcium/calmodulin-dependent protein kinase-stimulated binding of 14-3-3 to histone deacetylase 5. Proc. Natl. Acad. Sci. USA 97, 14400–14405 (2000). 11114197 10.1073/pnas.260501497 PMC18930

[b11] Vega R. B. . Protein kinases C and D mediate agonist-dependent cardiac hypertrophy through nuclear export of histone deacetylase 5. Mol. Cell. Biol. 24, 8374–8385 (2004). 15367659 10.1128/MCB.24.19.8374-8385.2004 PMC516754

[b12] Dequiedt F. . New role for hPar-1 kinases EMK and C-TAK1 in regulating localization and activity of class IIa histone deacetylases. Mol. Cell. Biol. 26, 7086–7102 (2006). 16980613 10.1128/MCB.00231-06 PMC1592903

[b13] Han J., Jiang Y., Li Z., Kravchenko V. V. & Ulevitch R. J. Activation of the transcription factor MEF2C by the MAP kinase p38 in inflammation. Nature 386, 296–299 (1997). 9069290 10.1038/386296a0

[b14] Kato Y. . BMK1/ERK5 regulates serum-induced early gene expression through transcription factor MEF2C. EMBO J. 16, 7054–7066 (1997). 9384584 10.1093/emboj/16.23.7054 PMC1170308

[b15] Sartorelli V., Huang J., Hamamori Y. & Kedes L. Molecular mechanisms of myogenic coactivation by p300: direct interaction with the activation domain of MyoD and with the MADS box of MEF2C. Mol. Cell. Biol. 17, 1010–1026 (1997). 9001254 10.1128/mcb.17.2.1010 PMC231826

[b16] Youn H. D., Grozinger C. M. & Liu J. O. Calcium regulates transcriptional repression of myocyte enhancer factor 2 by histone deacetylase 4. J. Biol. Chem. 275, 22563–22567 (2000). 10825153 10.1074/jbc.C000304200

[b17] Lilly B. . Requirement of MADS domain transcription factor D-MEF2 for muscle formation in Drosophila. Science 267, 688–693 (1995). 7839146 10.1126/science.7839146

[b18] Bour B. A. . Drosophila MEF2, a transcription factor that is essential for myogenesis. Genes Dev. 9, 730–741 (1995). 7729689 10.1101/gad.9.6.730

[b19] Edmondson D. G., Lyons G. E., Martin J. F. & Olson E. N. Mef2 gene expression marks the cardiac and skeletal muscle lineages during mouse embryogenesis. Dev. 120, 1251–1263 (1994). 10.1242/dev.120.5.1251 8026334

[b20] Potthoff M. J. . Regulation of skeletal muscle sarcomere integrity and postnatal muscle function by Mef2c. Mol. Cell. Biol. 27, 8143–8151 (2007). 17875930 10.1128/MCB.01187-07 PMC2169182

[b21] Hinits Y. & Hughes S. M. Mef2s are required for thick filament formation in nascent muscle fibres. Dev. 134, 2511–2519 (2007). 10.1242/dev.007088 PMC3016612 17537787

[b22] Potthoff M. J. . Histone deacetylase degradation and MEF2 activation promote the formation of slow-twitch myofibers. J. Clin. Invest. 117, 2459–2467 (2007). 17786239 10.1172/JCI31960 PMC1957540

[b23] Cohen T. J. . HDAC4 regulates muscle fiber type-specific gene expression programs. Mol. Cells 38, 343–348 (2015). 25728750 10.14348/molcells.2015.2278 PMC4400309

[b24] Cohen T. J. . The deacetylase HDAC4 controls myocyte enhancing factor-2-dependent structural gene expression in response to neural activity. FASEB J. 23, 99–106 (2009). 18780762 10.1096/fj.08-115931 PMC2626618

[b25] Rokach O. . Epigenetic changes as a common trigger of muscle weakness in congenital myopathies. Hum. Mol. Genet. 24, 4636–4647 (2015). 26019235 10.1093/hmg/ddv195

[b26] Cenik B. K. . Severe myopathy in mice lacking the MEF2/SRF-dependent gene leiomodin-3. J. Clin. Invest. 125, 1569–1578 (2015). 25774500 10.1172/JCI80115 PMC4396495

[b27] Shankar S. R. . G9a, a multipotent regulator of gene expression. Epigenetics 8, 16–22 (2013). 23257913 10.4161/epi.23331 PMC3549875

[b28] Shinkai Y. & Tachibana M. H3K9 methyltransferase G9a and the related molecule GLP. Genes Dev. 25, 781–788 (2011). 21498567 10.1101/gad.2027411 PMC3078703

[b29] Mozzetta C., Pontis J. & Ait-Si-Ali S. Functional Crosstalk Between Lysine Methyltransferases on Histone Substrates: The Case of G9A/GLP and Polycomb Repressive Complex 2. Antioxid. Redox Signal. 22, 1365–1381 (2015). 25365549 10.1089/ars.2014.6116 PMC4432786

[b30] Tachibana M. . G9a histone methyltransferase plays a dominant role in euchromatic histone H3 lysine 9 methylation and is essential for early embryogenesis. Genes Dev. 16, 1779–1791 (2002). 12130538 10.1101/gad.989402 PMC186403

[b31] Schaefer A. . Control of cognition and adaptive behavior by the GLP/G9a epigenetic suppressor complex. Neuron 64, 678–691 (2009). 20005824 10.1016/j.neuron.2009.11.019 PMC2814156

[b32] Ling B. M. T. . Lysine methyltransferase G9a methylates the transcription factor MyoD and regulates skeletal muscle differentiation. Proc. Natl. Acad. Sci. USA 109, 841–846 (2012). 22215600 10.1073/pnas.1111628109 PMC3271886

[b33] Choi J. . Modulation of lysine methylation in myocyte enhancer factor 2 during skeletal muscle cell differentiation. Nucleic Acids Res. 42, 224–234 (2014). 24078251 10.1093/nar/gkt873 PMC3874188

[b34] Rao V. K. . G9a promotes proliferation and inhibits cell cycle exit during myogenic differentiation. Nucleic Acids Res. doi: 10.1093/nar/gkw483 (2016). PMC5041453 27229136

[b35] Vedadi M. . A chemical probe selectively inhibits G9a and GLP methyltransferase activity in cells. Nat. Chem. Biol. 7, 566–574 (2011). 21743462 10.1038/nchembio.599 PMC3184254

[b36] Wang H. . NF-kappaB mediates the transcription of mouse calsarcin-1 gene, but not calsarcin-2, in C2C12 cells. BMC Mol. Biol. 8, 19 (2007). 17341303 10.1186/1471-2199-8-19 PMC1828060

[b37] Miska E. A. . Differential localization of HDAC4 orchestrates muscle differentiation. Nucleic Acids Res. 29, 3439–3447 (2001). 11504882 10.1093/nar/29.16.3439 PMC55849

[b38] McKinsey T. A., Zhang C. L., Lu J. & Olson E. N. Signal-dependent nuclear export of a histone deacetylase regulates muscle differentiation. Nature 408, 106–111 (2000). 11081517 10.1038/35040593 PMC4459600

[b39] Battisti V. . Unexpected distinct roles of the related histone H3 lysine 9 methyltransferases G9a and G9a-Like Protein in myoblasts. J. Mol. Biol. 428, 2329–2343 (2016). 27056598 10.1016/j.jmb.2016.03.029

[b40] Estrella N. L. & Naya F. J. Transcriptional networks regulating the costamere, sarcomere, and other cytoskeletal structures in striated muscle. Cell. Mol. Life Sci. 71, 1641–1656 (2014). 24218011 10.1007/s00018-013-1512-0 PMC3984630

[b41] Shi Y. . Coordinated histone modifications mediated by a CtBP co-repressor complex. Nature 422, 735–738 (2003). 12700765 10.1038/nature01550

[b42] Fritsch L. . A subset of the histone H3 lysine 9 methyltransferases Suv39h1, G9a, GLP, and SETDB1 participate in a multimeric complex. Mol. Cell 37, 46–56 (2010). 20129054 10.1016/j.molcel.2009.12.017

[b43] Ha C. H. . PKA phosphorylates histone deacetylase 5 and prevents its nuclear export, leading to the inhibition of gene transcription and cardiomyocyte hypertrophy. Proc. Natl. Acad. Sci. USA 107, 15467–15472 (2010). 20716686 10.1073/pnas.1000462107 PMC2932618

[b44] Klamut H. J., Bosnoyan-Collins L. O., Worton R. G. & Ray P. N. A muscle-specific enhancer within intron 1 of the human dystrophin gene is functionally dependent on single MEF-1/E box and MEF-2/AT-rich sequence motifs. Nucleic Acids Res. 25, 1618–1625 (1997). 9092671 10.1093/nar/25.8.1618 PMC146611

[b45] Oh S. Y. . The Histone Methyltransferase Inhibitor BIX01294 Inhibits HIF-1α Stability and Angiogenesis. Mol. Cells 38, 528–534 (2015). 26013382 10.14348/molcells.2015.0026 PMC4469910

[b46] Chen X., El Gazzar M., Yoza B. K. & McCall C. E. The NF-kappaB factor RelB and histone H3 lysine methyltransferase G9a directly interact to generate epigenetic silencing in endotoxin tolerance. J. Biol. Chem. 284, 27857–27865 (2009). 19690169 10.1074/jbc.M109.000950 PMC2788836

[b47] Han P. . Epigenetic response to environmental stress: Assembly of BRG1-G9a/GLP-DNMT3 repressive chromatin complex on Myh6 promoter in pathologically stressed hearts. Biochim. Biophys. Acta doi: 10.1016/j.bbamcr.2016.03.002 (2016). PMC7397641 26952936

[b48] Siegert R. . A myomesin mutation associated with hypertrophic cardiomyopathy deteriorates dimerisation properties. Biochem. Biophys. Res. Commun. 405, 473–479 (2011). 21256114 10.1016/j.bbrc.2011.01.056

[b49] Chiu C. . Mutations in alpha-actinin-2 cause hypertrophic cardiomyopathy: a genome-wide analysis. J. Am. Coll. Cardiol. 55, 1127–1135 (2010). 20022194 10.1016/j.jacc.2009.11.016

[b50] Osio A. . Myozenin 2 is a novel gene for human hypertrophic cardiomyopathy. Circ. Res. 100, 766–768 (2007). 17347475 10.1161/01.RES.0000263008.66799.aa PMC2775141

[b51] Frey N. . Mice lacking calsarcin-1 are sensitized to calcineurin signaling and show accelerated cardiomyopathy in response to pathological biomechanical stress. Nat. Med. 10, 1336–1343 (2004). 15543153 10.1038/nm1132

[b52] Chen G. . H3K9 histone methyltransferase G9a ameliorates dilated cardiomyopathy via the downregulation of cell adhesion molecules. Mol. Med. Rep. 11, 3872–3879 (2015). 25607239 10.3892/mmr.2015.3218

[b53] van Oort R. J. . MEF2 activates a genetic program promoting chamber dilation and contractile dysfunction in calcineurin-induced heart failure. Circulation 114, 298–308 (2006). 16847152 10.1161/CIRCULATIONAHA.105.608968

[b54] Xu J. . Myocyte enhancer factors 2A and 2C induce dilated cardiomyopathy in transgenic mice. J. Biol. Chem. 281, 9152–9162 (2006). 16469744 10.1074/jbc.M510217200

[b55] Sun H. . Stra13 regulates satellite cell activation by antagonizing Notch signaling. J. Cell Biol. 177, 647–657 (2007). 17502421 10.1083/jcb.200609007 PMC2064210

[b56] Nishio H. & Walsh M. J. CCAAT displacement protein/cut homolog recruits G9a histone lysine methyltransferase to repress transcription. Proc. Natl. Acad. Sci. USA 101, 11257–11262 (2004). 15269344 10.1073/pnas.0401343101 PMC509191

[b57] Shen H. . The Notch coactivator, MAML1, functions as a novel coactivator for MEF2C-mediated transcription and is required for normal myogenesis. Genes Dev. 20, 675–688 (2006). 16510869 10.1101/gad.1383706 PMC1413284

[b58] Wang X., Spandidos A., Wang H. & Seed B. PrimerBank: a PCR primer database for quantitative gene expression analysis, 2012 update. Nucleic Acids Res. 40, D1144–D1149 (2012). 22086960 10.1093/nar/gkr1013 PMC3245149

