# Supplementary material for: G9a inhibits MEF2C activity to control sarcomere assembly
Source: Sci Rep. 2016 Sep 26;6:34163. doi: 10.1038/srep34163 (PMC5036183; doi:10.1038/srep34163)
Supplement: Supplementary Information [file srep34163-s1.pdf]

G9a inhibits MEF2C activity to control sarcomere assembly

Jin Rong Ow, Monica Palanichamy Kala, Vinay Kumar Rao, Min Hee Choi, Narendra Bharathy and Reshma Taneja

SUPPLEMENTARY INFORMATION

Supplementary Figure S1

Supplementary Figure S2

Supplementary Figure Legends

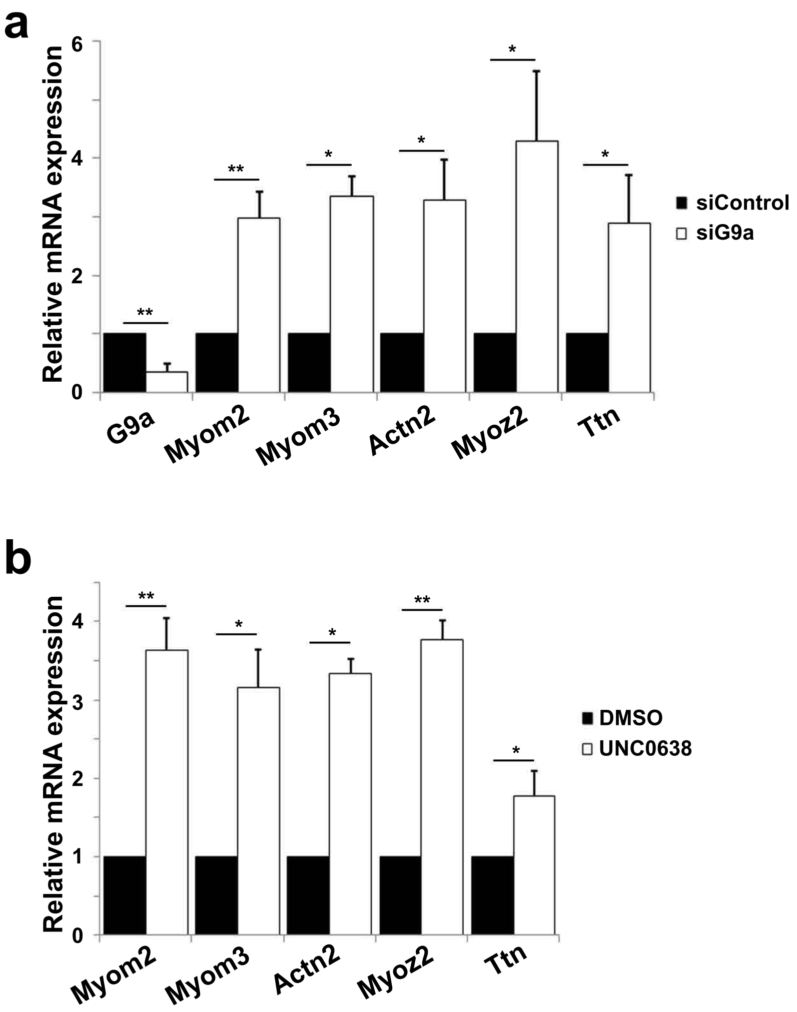
a
Relative mRNA expression
0
2
4
6
**
**
*
*
*
*
G9a
Myom2
Myom3
Actn2
Myoz2
Ttn
siControl
siG9a
b
Relative mRNA expression
0
1
2
3
4
**
*
*
**
*
Myom2
Myom3
Actn2
Myoz2
Ttn
DMSO
UNC0638

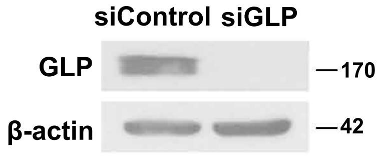
siControl siGLP
GLP
β-actin
170
42

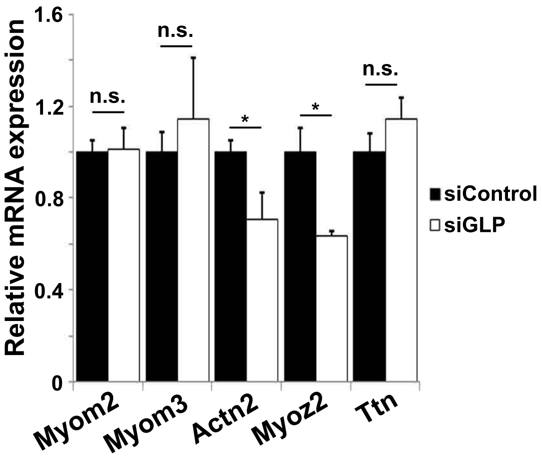
Relative mRNA expression
1.6
1.2
0.8
0.4
0
n.s.
n.s.
*
*
n.s.
Myom2
Myom3
Actn2
Myoz2
Ttn
siControl
siGLP

Supplementary Figure Legends

Supplementary Figure 1. G9a represses sarcomeric genes in primary myoblasts in a methyltransferase-dependent manner. **(a)** Primary mouse myoblasts transfected with scrambled siRNA (siControl) or G9a-specific siRNA (siG9a) lysates were analyzed for expression of G9a, Myom2, Myom3, Actn2, Myoz2 and Ttn by qPCR. Error bars indicate mean ±standard error. **(b)** Primary mouse myoblasts were treated with DMSO or UNC0638 for 48 hr in growth medium. Expression of sarcomeric genes in the absence and presence of UNC0638 treatment was examined by qPCR. Error bars indicate mean ±standard deviation; results are representative of two independent experiments. * p-value < 0.05; ** p-value < 0.01

Supplementary Figure 2. GLP does not regulate sarcomeric genes in myoblasts. C2C12 myoblasts transfected with scrambled siRNA (siControl) or GLP-specific siRNA (siGLP) were analyzed for GLP expression by western blot. β-actin was analyzed as a loading control. The mRNA expression of Myom2, Myom3, Actn2, Myoz2 and Ttn was analyzed by qPCR. Error bars indicate mean ±standard deviation; results are representative of two independent experiments. n.s. indicates no significance; * p-value < 0.05
